# Supplementary material for: RNAi-Mediated Functional Analysis Reveals the Regulation of Oocyte Vitellogenesis by Ecdysone Signaling in Two Coleoptera Species
Source: Biology (Basel). 2023 Sep 26;12(10):1284. doi: 10.3390/biology12101284 (PMC10604093; doi:10.3390/biology12101284)
Supplement: Supplementary file 1 [file biology-12-01284-s001.zip › biology-2516884-supplementary.pdf]

Supplementary data

RNAi-Mediated Functional Analysis Reveals the Regulation of Oocyte Vitellogenesis by Ecdysone Signaling in Two Coleoptera Species

Xiaoqing Zhang, Lin Jin, Guoqing Li*

Education Ministry Key Laboratory of Integrated Management of Crop Diseases and Pests/ State & Local Joint Engineering Research Center of Green Pesticide Invention and Application, Department of Entomology, College of Plant Protection, Nanjing Agricultural University, Nanjing 210095, China

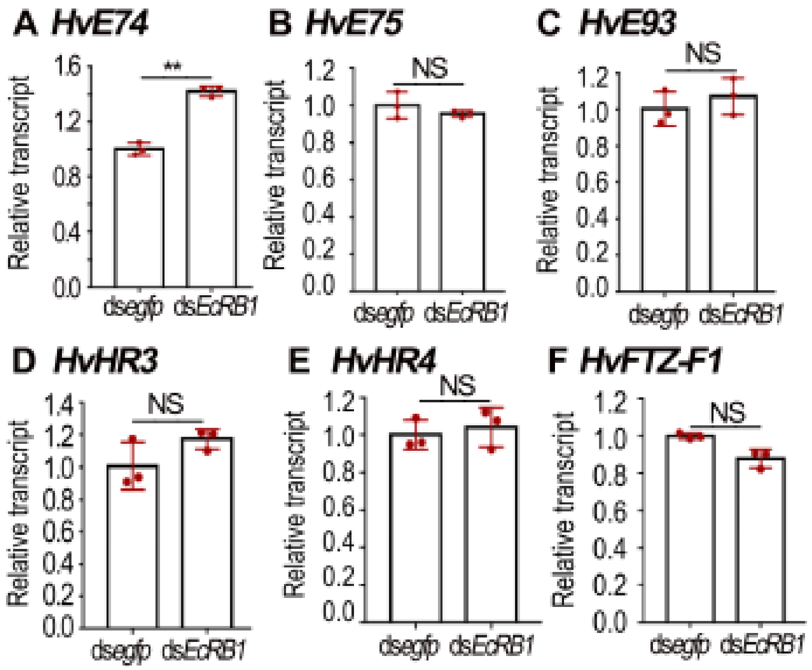

Figure S1. Knocking down *HvEcRB1* did not reduce the expression of 20E signaling genes in female adults. The newly emerged female adults were treated with 0.1 μL ds*egfp* and ds*EcRB1* (400 ng) by injection. The treated beetles were fed on fresh potato foliage. Ten days after treatment, transcript levels of six 20E signaling genes (*HvE74*, *HvE75*, *HvE93*, *HvHR3*, *HvHR4* and *HvFTZ*-F1) in ovaries were determined (A-F). Relative transcripts are the ratios of relative copy numbers in treated individuals to ds*egfp*-treated controls, which are set as 1. Different letters indicate significant difference at p value < 0.01 (**) using t-test. The columns represent averages with vertical lines indicating SD. NS, no significance.

Table S1. Primers used in RT-PCR, dsRNA synthesis and qPCR

Fragment name	Forward primer	Reverse primer
**RT-PCR**		
HvEcRA	GTCAAGCGAACGAAGGAAA	ATCCCGGATTTTAAATCCA
HvEcRB1	CCGTGTTTTCTCTCGGTCT	ATCCCGGATTTTAAATCCA
Hvusp1	ATGCCCTTCATTGACAACGAC	TCACAAACAACGTGGTCTAGTTGAA
Hvusp2	CTGAATACAATGAGTCCTCAGTCCC	CTTTTTCTCGGAGCTGGGTGA
LdEcRA	ACTCTCCCCAGTTACCAT	TCAGTCCAATCCAGTCTTA
LdEcRB1	GTTGTGGACGAAGAGTGA	CCGTCGAGTGTTAGTAGTAA
Ldusp1	ATGATGAAAAAGGAGAAGC	CTAAGTATCCGACTGGTTT
Ldusp2	ATGGGTCCGTTGGGTCCCC	CTAAGTATCCGACTGGTTT
**dsRNA synthesis**		
ds*HvEcR*	ACAAAGTGATGTCAGATTTAG	GCATTATCTACTTTCATGTAA
ds*HvEcRB1*	GTCTCAGTAGCGCGATTT	GGAGACGGATATCCATTAC
ds*Hvusp*	CTCTCGGAAATTGTAAATAAAA	CCGTTAGAAAATTATCGATACT
ds*LdEcRA*	GCAGCATGGACATCAAACAC	GCACTTACACTAATGGCTCCC
ds*LdEcRB1*	GATGGAACGGCTTCAGGG	CACTTTTAGCCAATGTATGACTG
ds*Ldusp-1*	AATCAATAAGTCCACCGC	CCAGACACTTCAAACCGA
ds*Ldusp-2*	CGTATCCTCCCAACCATC	CTCTCTTCTCGGCTTCCA
ds*egfp*	AAGTTCAGCGTGTCCG	CACCTTGATGCCGTTC
**qPCR**		
qHvVg	CGTTGGTGAAAGGACGGAGA	CTCGGACAGCATGATTGGGT
qHvVgR	TGCAACAACGGCAAATGTAT	GCCGACATTGGTAAGCAAAT
qHvEcR	AGAAGAAAGCCTAGGTCCGG	GTCCCATATCTCGGCCAAGA
qHvEcRA	GCGTAGATGGACGGGTATGC	AACGTCGCAGACGAAGATGT
qHvEcRB1	GCAGCTCCAATCTCAATGGT	GAATTCGCGTTCTGGTTGTT
qHvusp	GGATGCATCAGAGGTGGAGT	AAACTTGGTCACACACTGGC
qHvE93	GCTCATCCGACCTTTCACCA	TCTTGGGTAGCCGGGTATGA
qHvFTZ-F1	CACATCGACAAGACGCAGAG	ACAGCTTCGAGTTTCATGCC
qHvE74	AACCGTCGTTACATTCGCCT	TTGCTGAGGTATGTGGCCTG
qHvE75	TAAAAAGTTCCGCGCGCTTC	GTTGGGGTTGCGGAGACATA
qHvHR3	CACGACTACCTACGACCCAA	TGAAACGCTCGCTATGATGC
qHvHR4	CACGACGTCGGACTGATGAT	GGCACGTCATATACCGCTCA
qHvRPS18	CGCAATCAAAGGTGTTGGAAG	GCCTAGGGTTGGCCATAATAG
qHvRPL13	AGCATCCTTCGCTCGTTTAG	TTCGACAACCTGCCATTAGG
qLdEcRA	CGGGTTCAATAACAGTGTCG	ATAAGGTTGCGAAGGTGGTC
qLdEcRB1	GGGAGTGCTAGTGTTGTGGA	GGATTAGACGCTCCCTACGA
qLdusp	GTACTGGCATCTGTTTCTTTGTCG	CAAGAAGAACGGCAAAGGACGA
qLdVg1	AGTCGTGCTGTTCTCACAGG	CTTCCTCCAGGGTGACGTTC
qLdVg2	ATGCTGAGCAAGGTCCGAAA	GTTCACGAATGGCGTTAGCC
qLdVgR	CTTCAGCCGAGTGTGGGTAA	TAGAAGCGTTCCATCCTGGC
qLdInR1	TCTCAAGCGAATCAGAGGTG	GTTACGCCAATCCCAAAGTT
qLdFTZ-F1	GGCTAATCAGGCCTCCAG	CATGGTTTGCTGGCAACTAC

qLdARF1	CGGTGCTGGTAAAACGACAA	TGACCTCCCAAATCCCAAAC
qLdRP18	TAGAATCCTCAAAGCAGGTGGCGA	AGCTGGACCAAAGTGTTTCACTGC
qLdRP4	AAAGAAACGAGCATTGCCCTTCCG	TTGTCGCTGACACTGTAGGGTTGA
qLdARF4	GTGCTCGTGAACCATGTGAA	AACCTCCAATCCCTCGTGAA
